# A genomics approach in determining nanotopographical effects on MSC phenotype

**DOI:** 10.1016/j.biomaterials.2012.12.019

**Published:** 2013-03

**Authors:** Penelope M. Tsimbouri, Kate Murawski, Graham Hamilton, Pawel Herzyk, Richard O.C. Oreffo, Nikolaj Gadegaard, Matthew J. Dalby

**Affiliations:** a Centre for Cell Engineering, Institute of Molecular, Cell and Systems Biology, College of Medical, Veterinary and Life Sciences, Joseph Black Building, University of Glasgow, Glasgow G12 8QQ, Scotland, UK; b Division of Biomedical Engineering, School of Engineering, University of Glasgow, Glasgow G12 8LT, Scotland, UK; c Sir Henry Wellcome Functional Genomics Facility, College of Medical, Veterinary and Life Sciences, Joseph Black Building, University of Glasgow, Glasgow G12 8QQ, Scotland, UK; d Bone & Joint Research Group, Centre for Human Development, Stem Cells and Regeneration, Institute of Developmental Sciences, University of Southampton, Southampton SO16 6YD, UK; e Stem Cell Unit, Department of Anatomy, College of Medicine, King Saud University, Riyadh, Saudi Arabia

**Keywords:** Mesenchymal stem cells (MSCs), Nanotopography, Mechanotransduction, Gene expression, Cell signalling, Molecular biology

## Abstract

Topography and its effects on cell adhesion, morphology, growth and differentiation are well documented. Thus, current advances with the use of nanotopographies offer promising results in the field of regenerative medicine. Studies have also shown nanotopographies to have strong effects on stem cell self-renewal and differentiation. What is less clear however is what mechanotransductive mechanisms are employed by the cells to facilitate such changes. In fastidious cell types, it has been suggested that direct mechanotransduction producing morphological changes in the nucleus, nucleoskeleton and chromosomes themselves may be central to cell responses to topography. In this report we move these studies into human skeletal or mesenchymal stem cells and propose that direct (mechanical) signalling is important in the early stages of tuning stem cell fate to nanotopography. Using fluorescence *in situ* hybridization (FISH) and Affymetrix arrays we have evidence that nanotopography stimulates changes in nuclear organisation that can be linked to spatially regulated genes expression with a particular focus on phenotypical genes. For example, chromosome 1 was seen to display the largest numbers of gene deregulations and also a concomitant change in nuclear positioning in response to nanotopography. Plotting of deregulated genes in reference to band positioning showed that topographically related changes tend to happen towards the telomeric ends of the chromosomes, where bone related genes are generally clustered. Such an approach offers a better understanding of cell–surface interaction and, critically, provides new insights of how to control stem cell differentiation with future applications in areas including regenerative medicine.

## 1 Introduction

Current developments in the application of nanotopography have provided us with promising results in the field of regenerative medicine. Major results with mesenchymal stem cells, a key regenerative cell target given their indicated immune-privilege and availability as autologous cells, have included the ability to target osteogenesis using controlled disorder, NSQ50 (pits of 120 nm diameter, 100 nm deep with a near square arrangement – average 300 nm centre–centre with up to ±50 nm offset in X and Y) indicating that implant modifications may be possible to improve clinical outcome [1–4]. More recently, it was shown that nanostructured surfaces with tightly controlled arrangement, SQ (similar to NSQ50 but with no offset) can retain stem cell phenotype and maintain stem cell growth with implications therein for provision of high quality stem cells to clinic [5]. Furthermore, recent literature has also highlighted the potential for modifying embryonic stem cell response with nanotopography [6,7].

Understanding the mechanism of the physiological processes that control cell–biomaterial interactions and the influence of nanotopography on cell adhesion and phenotype is fundamental to understanding stem cell differentiation. In this study isolated multipotential bone marrow skeletal stem cells also known as mesenchymal stem cells (MSCs), with the potential to differentiate along the stromal lineages, were examined. MSCs can give rise to different lineages including fibroblastic, chondrogenic, myoblastic, adipogenic, and osteoblastic cell types [8–11]. Recent studies have highlighted that MSC function follows form, with alterations in cell adhesion and subsequent cytoskeletal tension modulating lineage commitment [9,12]. There is evidence demonstrating the importance of intracellular tension in MSCs with a high-tension state inducing osteogenic differentiation, whilst a low-tension state inducing adipogenic differentiation [12–14]. Recent advances are indicative of the requirement of an intermediate level of cellular tension for MSC self-renewal [5,15,16].

Interactions between stem cells and the ECM can have indirect or direct effects on cells, otherwise known as mechanotransduction, to elicit changes in gene expression.

Indirect mechanotransduction includes the canonical biochemical signalling cascades which result from integrin binding and focal adhesion formation [17]. The second form of mechanotransduction, direct, occurs as a consequence of conformational changes in the cell cytoskeleton, which forms a direct link between the extracellular matrix and the nucleus of the cell via the nucleoskeletal lamins (the intermediate filaments of the nucleus) and potentially further to the chromosomes via telomeric chromatin/lamin interactions [18,19].

The nucleus itself is supported by the nucleoskeleton consisting of a network of proteins comprising lamins A and C (derived by differential splicing of the same gene) [20], B1 [21] and B2 [22] (products of two genes) in most somatic cell types. The lamina provides structural support to the nucleus, forming part of the link to the cytoskeleton ensuring the correct nuclear and centrosomal organization. The lamina is also associated with DNA replication; lamin B foci are associated with proliferating cell nuclear antigen (PCNA), a protein acting as a processivity factor for DNA polymerase δ in eukaryotic cells creating topological links to the genome during DNA replication [23,24]. Nanotopography produces tension related changes in-line with the literature for stiffness and chemistry. For example, high-tension for MSCs on NSQ50 nanotopographies resulting in osteogenesis and intermediate tension for MSCs on SQ nanotopographies supports self-renewal [5,16]. We also understand that these tension states drive indirect cascades such as extracellular-signal-regulated kinases, (ERK1/2), c-Jun N-terminal kinase (Jnk) and low density lipoprotein (LDL) signalling [16]. However, what is less clear is whether these tension states drive direct changes in nuclear architecture and if there is a possible link to phenotype arising from such changes.

To address the role of direct mechanotransduction on MSC differentiation, the SQ (self-renewal promoting) and NSQ50 (osteogenesis promoting) nanotopographies were employed in these studies. We have examined the nucleus and have used fluorescence *in situ* hybridisation (FISH) to study movement of chromosomes in the MSCs on the defined nanotopographies. Chromosome choice was informed by microarray analysis implicating the chromosomes with the greatest expression profile change. In addition, using the gene expression data, spatial activity along the chromosomal arms was examined and gene and protein level data on key transcription factors for differentiation and phenotypical markers for MSC phenotype were linked to these spatial ‘bins’. The experiments were performed after three days of culture in order to capture morphological changes in the early stages of cell decision making in maintaining self-renewal or starting to express early differentiation-related transcription factors.

## 2 Materials and methods

### 2.1 Nanopatterning and mastering

The substrates were made in a three-step process of electron beam lithography [25] nickel die fabrication and polymer replication using injection moulding. Briefly, the master substrates were fabricated to form an array of 120 nm diameter pits of 100 nm depth and 300 nm pitch in a square (SQ) arrangement with the near square (NSQ50) substrate has a random displacement of ±50 nm, and maintaining an average 300 nm pitch. Nickel dies were made directly from the patterned resist samples and a thin (50 nm) layer of Ni–V was sputter coated on the samples, acting as an electrode in the subsequent electroplating process. The dies were plated to a thickness of approximately 300 μm. The nickel shims were cleaned by stripping the protective polyurethane coating using chloroform in an ultrasound bath for 15 min. An injection moulder was used to make polymer replicas in polycarbonate.

### 2.2 Cell extraction and culture

MSCs or skeletal stem cells were enriched from human bone marrow using the STRO1 antibody and magnetic activated cell sorting (MACS) as previously described [2]. MSCs were maintained in basal media (αMEM (PAA)) supplemented with 10% FBS (PAA), 1% (v/v) 200 mm l-glutamine (Gibco) and antibiotics (6.74 U/ml Penicillin–Streptomycin, 0.2 μg/ml Fungizone, Gibco) at 37 °C with 5% CO_2_ in a humidified incubator. MSCs were seeded onto the materials at 1 × 10^4^ cells/ml and allowed to grow for 3 days. Cells were used at passages P1–P2 throughout the study. Cells were isolated from a large number of patients (>10) and were used over the course of the studies to help show robustness of the data.

### 2.3 Chromosome territory staining: fluorescence *in situ* hybridisation (FISH)

MSCs were fixed in 3:1 methanol:acetic acid for 30 min at room temperature and rinsed in 2× SSC (saline sodium citrate; diluted from 20× stock of 3 m NaCl, 0.3 m *tri*-sodium citrate, pH7.4) for 3 h at 37 °C. The appropriate chromosome probe (biotinylated human chromosome 1 paint; Cambio, Cambridge, UK) was brought to 37 °C, vortexed, and pelleted by centrifugation for ∼3 s at 11,000 × *g*. The probe was denatured at 65 °C for 10 min, followed by a 30 min incubation at 37 °C. The samples were rinsed in H_2_O for 30 s and then dehydrated through a 70%, 90%, 90% (v/v) ethanol series, with a 2 min incubation at each step, followed by a 5 min dehydration step in 100% ethanol. The samples were then air dried for 1 min and incubated in denaturation solution (7:1 formamide: 2× SSC buffer) at 65 °C for 2 min. The samples were quenched using an ice-cold ethanol series as above and air-dried for 1 min. The denatured probe (8–15 μl) was added to each sample, the samples were covered with coverslips and incubated for 44 h at 37 °C in a humidified chamber. Following hybridization, the samples were rinsed in 45 °C pre-warmed 1× SSC buffer for 5 min followed by 2 × 5 min washes in stringency wash solution (1:1 formamide: 1× SSC). The probe was detected using the Biotin Painting Kit (Cambio), according to the manufacturer's protocol. Three replicates of each topography (NSQ50, SQ, FLAT) were used in each experiment.

### 2.4 Territory analysis

The distances from the nearest edge of the nuclei to the centres of the chromosomal territories and the interterritory distances were measured using Image J (version 1.34s; Rasband, W.S., Image J, U.S. National Institutes of Health – http://rsb.info.nih.gov/ij/). Statistics were generated using Prism (GraphPad at www.graphpad.com/prism) the Tukey–Kramer multiple comparisons post-test analysis of variance (ANOVA).

### 2.5 Affymetrix arrays

MSCs were cultured on the topographies (4 material replicates/biological replica/topography) for 3 days. At this point, the cells were lysed and total RNA was extracted using a Qiagen RNeasy micro kit (Qiagen, UK). Gene expression changes were detected by hybridization of mRNA to Affymetrix HuGene 1.0 ST human arrays according to the manufacturers instructions. Initial bioinformatic analysis was based on rank product. A false discovery rate of 20% was used to upload selected genes changes to the Ingenuity Pathway Analysis (IPA) server to identify canonical signalling pathways, functional pathways and to produce networks. Statistics for functional analysis were carried out by Fischer's exact test (automatically performed by the software). For the chromosomal band identification a custom script was written to add annotations to the ANOVA results file. The script generated a transcript cluster ID to the chromosome lookup table from the HuGene-1_0-st-v1.na32.hg19.probeset.csv file obtained from Affymetrix (www.affymetrix.com). Then the results file was parsed and the chromosomal location details, from the lookup table, were appended to the corresponding transcript.

### 2.6 Quantitative real time (q)PCR

MSCs were cultured on topographies for 3 days (4 biological replicas (each consisting of 3 replicas each pooled) for each NSQ50, SQ and FLAT) at a density of 1 × 10^4^ cells/ml. Total RNA was extracted using a Qiagen RNeasy micro kit. Real-time qPCR was carried out and analysed as previously described to assess the expression of Runx2, HOP26, ALCAM, SOX9 and PParγ (Tables 1 and 2). RNA samples were reverse transcribed using the Omniscript First Strand System (Qiagen). Real-time qPCR was carried out using the 7500 Real Time PCR system from Applied Biosystems. GapDH served as the house-keeping gene to normalise expression for the genes of interest. In cases where the SYBR Green method was used (Table 1), primer sequences for the genes (GapDH and ALCAM) were validated by dissociation curve/melt curve analysis. Alternatively, Applied Biosystems probes were used (Table 2) using the TaqMan FAST Universal mastermix. The GapDH housekeeping gene primer/probe set was used (ABI predesigned amplification reagent) for normalisation and primer and FAM-MGB probe sets were used for SOX9, Runx2, PParγ and Hop26. The $2^{-\Delta \Delta C_{\text{T}}}$ method [26] was used for analysis of gene expression. Statistical analysis was carried out using the Tukey–Kramer multiple-comparisons post-test analysis of variance (ANOVA). The relative transcript levels were expressed as the mean ± s.d. (*n* = 3 for each group) for plotting as a graph.

### 2.7 Western blotting

MSCs were cultured on the topographies for 3 days (4 biological replicas (each consisting of 4 replicas each pooled) for each NSQ50, SQ and FLAT) at a density of 1 × 10^4^ cells/ml. MSCs were lysed using protein lysis buffer (20 mm Tris–HCl, pH 7.5, 150 mm NaCl, 1 mm EDTA, 1% v/v Triton X-100) containing phosphatase and protease inhibitors (1% phosphatase inhibitor cocktail Roche cat. no. 0490684500, 1% Sigma protease inhibitor cocktail, cat. no. P2714). Proteins were run on a pre-cast NOVEX gradient (4–12%) gel system (Invitrogen) and the samples were then transferred onto a nylon membrane (Imobilon P, Millipore), according to the manufacturer's protocol. For probing, the blots were incubated in 5% non-fat milk PBS 0.1% (v/v) Tween 20 with the appropriate anti-sera dilution. Antibodies (with dilutions) used were directed to: osteogenic markers Phospho-Runx2 (Abgent, AP3559a) 1:500 total Runx2 (Santa Cruz Biotechnology, sc-10758) 1:500, stem cell marker STRO1 1:500 (R&D systems, MAB1038), adipocytic marker PParγ (Santa Cruz Biotechnology, sc-1984), chondrocytic marker SOX9 (Abcam, ab76997), housekeeping gene GapDH (Sigma, G8795); followed by the appropriate 1:4000 goat anti-rabbit, goat anti-mouse or donkey anti-goat IgG HRP-conjugates (Santa Cruz). Detection was performed by enhanced chemiluminescence (Immobilon Western, Millipore).

## 3 Results

### 3.1 Nuclear organization

**Fig. 1:**
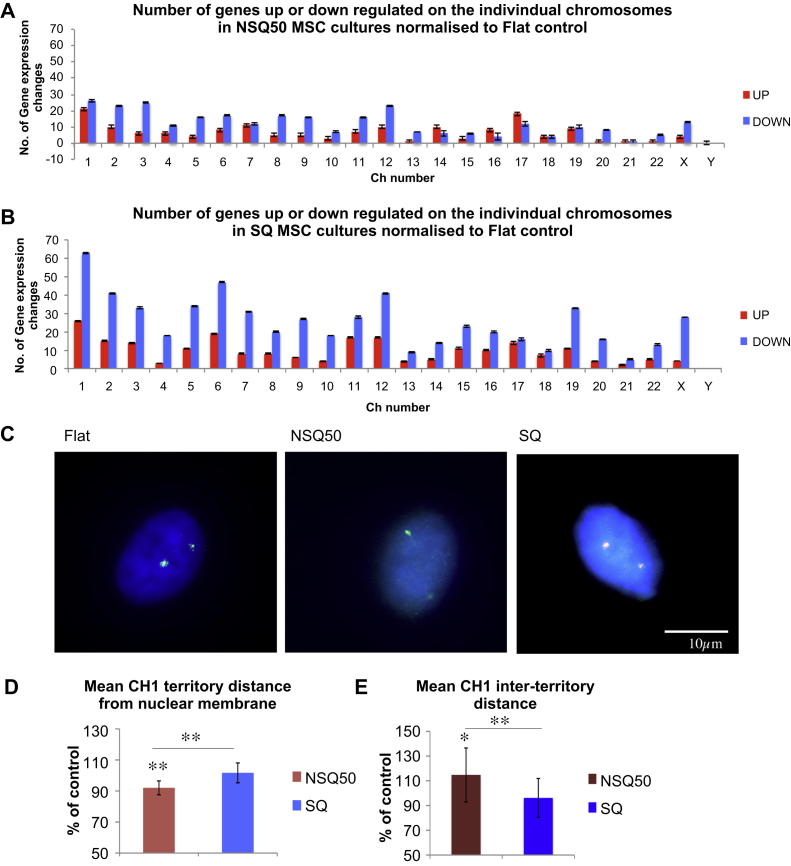
Nanotopography effects on chromosome 1 (Ch1) territory repositioning within the MSC nucleus. (A) and (B) Microarray was used to select chromosomes that showed either few or many transcript abundance changes. Examples NSQ50 (A) and SQ (B) MSC cultures. Ch1 had the greatest total number of expression changes (mostly down-regulated genes). (C) FISH staining for chromosomal territory positioning. Examples of FISH staining of Ch1 on flat, NSQ50 and SQ in MSC nuclei. Key: Blue – DNA; Green: Ch1 territories. (D) Chromosome territory positioning within the nucleus. For chromosome 1, quantification of territory position relative to the nuclear periphery was significantly altered (*p* < 0.01) for cells cultured on NSQ50 compared to those on the flat control and SQ. (E) For chromosome 1, quantification of inter-territory position was significantly larger (*p* < 0.05) for cells cultured on NSQ50 compared to those on the flat control and (*p* < 0.01) when compared to those on the SQ. Comparison was done by ANOVA *p < 0.05, **p < 0.01, ***p < 0.001, *n* = 45. (For interpretation of the references to colour in this figure legend, the reader is referred to the web version of this article.)

Microarray analysis was used to identify potential ‘hotspots’ for differential gene expression in the genome of MSCs grown on the different nanotopographies and to contribute to the rational selection of specific chromosomes. At the chromosomal level, chromosome 1 (Ch1) displayed the largest number of differentially expressed genes (up and down regulated) in cells cultured on both NSQ50 and SQ (Fig. 1A and B). Overall NSQ50 displayed the most changes (Supplementary Fig. 1) indicating the higher impact this topography has on MSC gene expression and cell growth. In general, both topographies displayed the most regulation changes on predominantly on the larger chromosomes (such as Ch1, Ch2, Ch5, Ch6, Ch12, and ChX) indicating a trend between size and response to nanotopography.

While Ch17 and Ch19, smaller chromosomes, showed deregulations in response to nanotopography, this could, speculatively, be attributed more to biochemical changes over the direct mechanotransduction.

Based on the above observations, Ch1 was investigated further by FISH in order to examine the involvement of chromosomal territory shifts on differential gene expression (Fig. 1C). Distances from the nearest edge of the nucleus to the centre of chromosomal territories were measured for Ch1. Quantification of Ch1 territory position relative to the nuclear periphery was significantly altered (*p* < 0.01, ANOVA) for cells cultured on the NSQ50 compared to those on the planar control and SQ (*p* < 0.01, ANOVA) although chromosomal territory position was comparable between SQ topography and Flat control (Fig. 1D). In addition, the Ch1 inter-territory distance in cells on the NSQ50 topography was statistically significantly larger (*p* < 0.05) compared to those on the Flat control and (*p* < 0.01) when compared to those on the SQ (Fig. 1E).

Further evidence supporting our hypothesis that topographical differences affect the nuclear physiology and organization with concomitant chromosomal shifts are observations of nucleus size differences between cells on the NSQ50 in comparison to cells on the SQ nanotopography and Flat control (Supplementary Fig. 2).

The microarray data was used to identify the chromosomal band positions of differentially expressed genes. Band positions were assayed using *Z*-score analysis. Z score measures the probability of the observed numbers of changes, occurring at a particular chromosomal band position with higher scores in either direction indicating that the observed number of gene expression changes are most likely true.

**Fig. 2:**
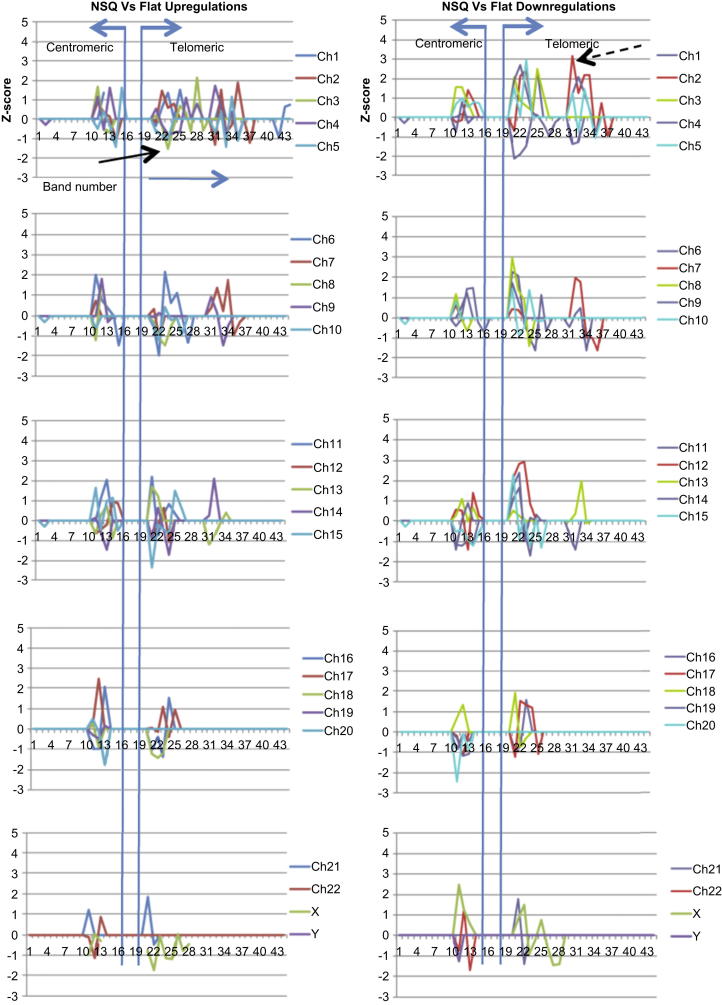
NSQ50 *Z*-score analysis of gene activity associated with q-arm band number. The graphs show significant differences from expected changes based on analysis of 1600 iterations of a ‘window’ of genes that were selected using a threshold of 15% for the false discovery rate. Data is shown NSQ50:Flat (up-regulations/down-regulations refer to the NSQ50 topography, relative to the Flat surface). For the largest chromosomes (1–5), analysis showed points of loss of up-regulation (→) and many more substantial gains of down-regulation () at the telomeric regions (band 20 and above). Profiles were similar for chromosomes 6–15. Interestingly, there were a large number of ‘gains’ of gene up-regulation associated with ‘loss’ of gene down-regulation at the centromeric regions (band 10–15) of chromosomes 16 and above.

**Fig. 3:**
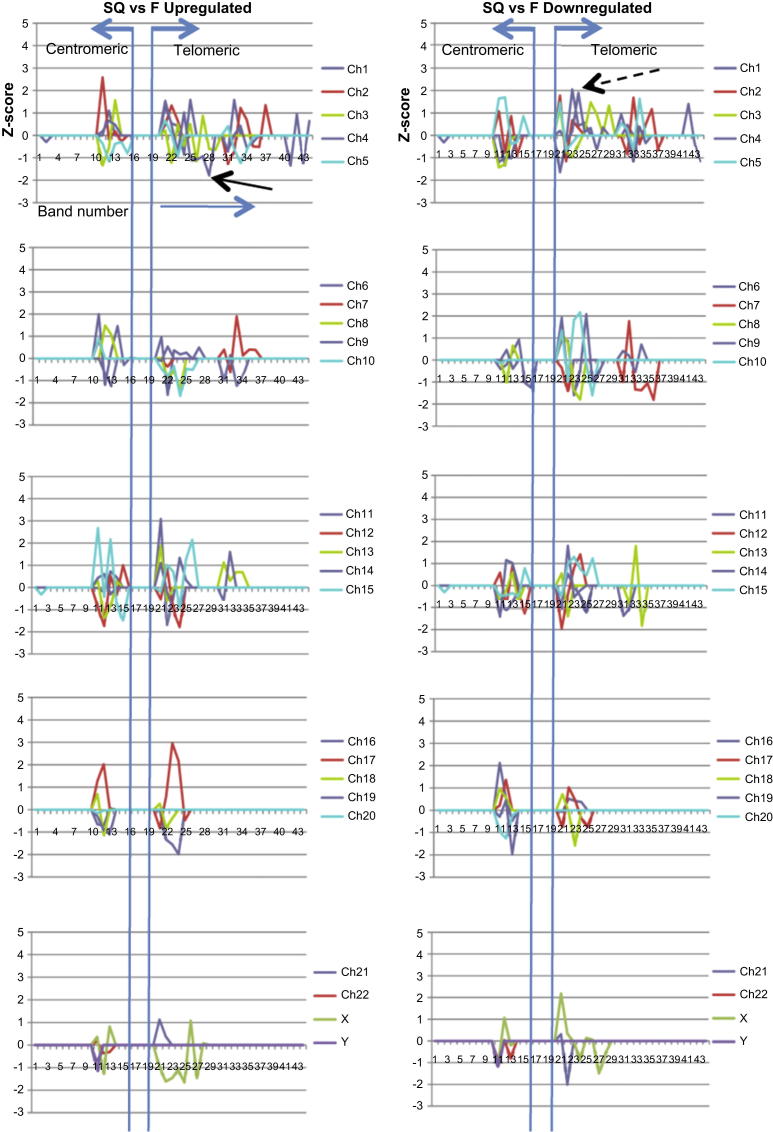
SQ *Z*-score analysis of gene activity associated with q arm band number. The graphs show significant differences from expected changes based on analysis of 1600 iterations of a ‘window’ of genes that were selected using a threshold of 15% for the false discovery rate. Data is shown SQ:Flat (up-regulations/down-regulations refer to the SQ topography, relative to the Flat surface). For the largest chromosomes (1–5), analysis showed points of loss of up-regulation (→) and many more substantial gains of down-regulation () at the telomeric regions (band 20 and above). Profiles were similar for chromosomes 6–10. Interestingly, there were a large number of ‘gains’ of gene down-regulation associated with ‘loss’ of gene up-regulation at the telomeric regions of chromosomes 11–15. Profiles were very similar for the small chromosomes (16–22).

The results from the q-arm data showed that the topography-related changes for both NSQ50 (Fig. 2) and SQ (Fig. 3) were broadly spaced along Ch1 with a trend of activity towards the telomeric (end) region of the chromosome. This was apparent for most of the larger chromosomes (1–15), where analysis showed points of loss of up-regulation and enhanced down-regulation at the telomeric regions (band 20 and above). Interestingly, there were a number of ‘gains’ of gene up-regulation associated with ‘loss’ of gene down-regulation at the centromeric regions (band 10–15) of chromosomes 16 and above.

Looking at the p-arm analysis results for MSCs on NSQ50 (Supplementary Fig. 3), for the largest chromosomes (1–5), analysis showed points of loss of up-regulation (→) and many more substantial gains of down-regulation () at predominantly the centromeric regions (bands 11–15) and less at the telomeric regions (band 21 and above). Similar profiles were obtained for chromosomes 6–10. Furthermore, there were a large number of ‘gains’ of gene down-regulation associated with ‘loss’ of gene up-regulation at the centromeric regions of chromosomes 11 and above.

SQ p-arm analysis results, for the largest chromosomes (1–5), showed points of loss of down-regulation (→) and many more substantial gains of up-regulation () at the telomeric regions (band 20 and above) (Supplementary Fig. 4). Interestingly, there were a large number of ‘gains’ of gene up-regulation associated with ‘loss’ of gene down-regulation at the centromeric regions (bands 10–15) of chromosomes 6–15. In addition, there were a large number of ‘gains’ of gene down-regulation associated with ‘loss’ of gene up-regulation at the centromeric regions of chromosomes 16 and above.

### 3.2 Correlating morphology to phenotype

**Fig. 4:**
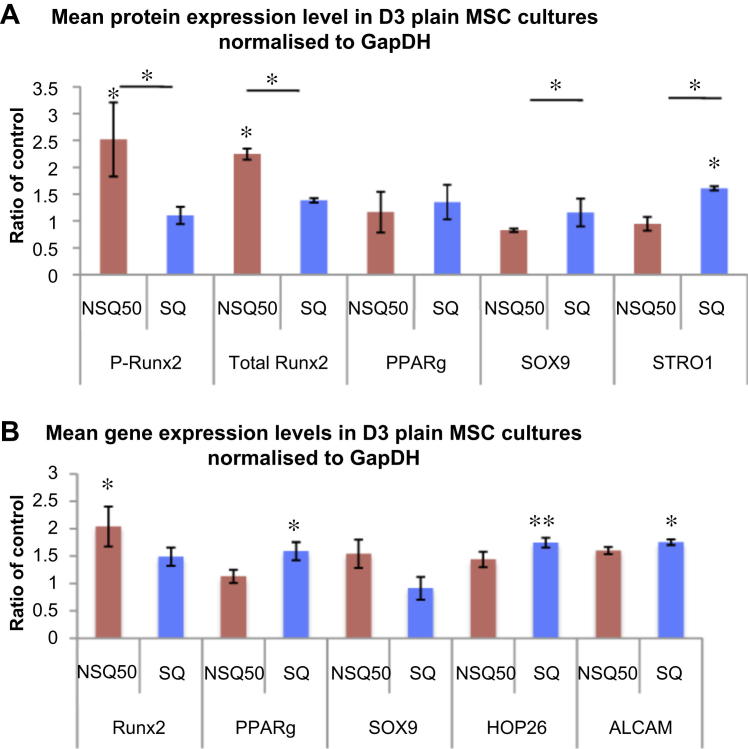
Phenotypic changes in response to nanotopography. A). Mean protein expression levels in MSCs grown on NSQ50 and SQ nanotopographies expressed as ratio of the flat control. B) Mean gene expression levels in MSCs grown on NSQ50 and SQ nanotopographies expressed as ratio of the flat control. NSQ50 (high tension) supported the expression of osteogenic marker Runx2 but not soft tissue marker such PPARγ, SOX9 and STRO1. In contrast, SQ (low tension) supported expression of soft tissue markers, especially STRO1 but not Runx2. Comparison was done by ANOVA *p < 0.05, **p < 0.01, ***p < 0.001, *n* = 3. Note: markings above the error bars denote comparison to flat control; comparison between SQ and NSQ50 is denoted by a line.

In an effort to correlate morphology to phenotype, protein and gene expression studies were performed. The mean protein expression levels in MSCs grown on NSQ50 (osteogenesis promoting) and SQ (self-renewal supporting) nanotopographies were investigated using Western blotting (Fig. 4A) and gene expression was studied by qPCR (Fig. 4B). The markers used, typically associated with multipotent skeletal or mesenchymal stem cells, were STRO1, HOP26, and ALCAM and differentiation was indicated by tissue specific markers Runx2 (runt related transcription factor 2, indicative of initiation of osteogenesis), soft-tissue markers SOX9 (Sry-related high mobility group box 9, a cartilage specific transcription factor) and PParγ (peroxisome proliferator-activated receptor gamma, an adipocyte-related transcription factor). NSQ50 supported the expression of the osteogenic marker Runx2 in both active (phosphorylated) and total protein forms (Fig. 4A) as well as at the gene transcript level (Fig. 4B) at considerably higher levels than for cells on flat controls and SQ topography (*p* < 0.05). Examination of soft tissue differentiation markers such as adipogenic marker PPARγ and bone stem cell marker STRO1 showed a reduction at both gene and protein levels of all these markers on the NSQ50 (osteogenic differentiation promoting topography) in comparison to SQ and Flat control (Fig. 4A and B). The chondrogenic marker SOX9 showed reduced gene (*p* < 0.05) levels in MSCs on NSQ50 with no significant change at protein level noted. For the SQ, self-renewal promoting, nanotopography, statistically significant (*p* < 0.01) increased protein levels of STRO1 and gene levels of HOP26 and ALCAM (primers cannot yet be designed for STRO1 in the absence of epitope data so HOP26 and ALCAM genes were used as alternatives) were observed in agreement with Refs. [5,16].

## 4 Discussion

In the field of regenerative medicine, understanding the stem cell nano/microenvironment is of primary importance. The elucidation of the cell–nanotopographical interaction and its effects on cell morphology and phenotype will provide new insights into the regulation of stem cell differentiation and self-renewal processes. It has previously been shown [1] that a slightly disordered nanotopography NSQ50 promotes osteogenic differentiation and, more recently, we demonstrated that a highly ordered nanotopography, SQ, supports the human skeletal stem cell phenotype [5].

Here, we confirm and extend these results with protein and gene expression analyses to study the differentiation status of MSCs on the two nanotopographies. NSQ50 supported expression of the osteogenic marker Runx2 (Fig. 4). This is in agreement with the previous studies where osteogenic markers, such as osteopontin and osteocalcin, were observed [1]. In contrast, the SQ topography supported the expression of skeletal stem cell enrichment markers STRO1, HOP26 and ALCAM (Fig. 4). The generation and availability of two surfaces with similar physical and chemical properties (contact angle and feature shape, size and coverage are identical – only pit arrangement changes) provides materials that serve as ideal controls with which to compare and contrast differentiation and self-renewal; in this study we have a focus on direct mechanotransduction. We note that other studies have implicated nanoscale order and disorder as having large effects on MSC attachment and adhesion based on ability to gather integrins into focal adhesions [27].

Cell–extracellular matrix or cell–surface interactions trigger cascades of signals via membrane proteins at points of adhesion that are transmitted through the cytoskeleton to the nucleus and determine stem cell homing, proliferation and differentiation processes [28]. It is known that as the cytoskeleton is linked to adhesions and also to the nucleoskeleton via LINC (linkers of nucleoskeleton to the cytoskeleton) complexes (possibly facilitated by tensegrity) that alterations in nucleus morphology will follow rearrangements of the cytoskeleton and we have produced preliminary evidence for this in fibroblasts [16,29–31]. It has been demonstrated by others that high tension and cell spreading are essential for the differentiation of MSCs to osteoblasts [9,13]. Here we postulate that the effects may be more than simply biochemical with direct application of tension to the nucleus (based also on nuclear morphology observations in Supplementary Fig. 2) and coupling of the chromosomal telomeres to the nuclear lamina resulting in changes in chromosomal positioning altering DNA accessibility to transcription factors. Such a mechanism of action has also been postulated in response to changes in serum concentration [32].

For the SQ, self-renewal, surface, much smaller (compared to NSQ50) differences to the flat control were seen in chromosomal positioning. Here, we observed expression of soft tissue markers such as SOX9 (mapped on chromosome 7p-arm band 21), PPARγ (12p13), HOP26 (17q11) that are mainly located on smaller chromosomes at more centromeric positions. We propose smaller chromosomes are less tension-affected as previous studies with fibroblasts also indicate that smaller chromosomes are less responsive to topography. In addition, it is important to note that it is the telomeres that are attached and thus mechanically connected to the nuclear lamina [1,31]. This is perhaps suggestive of more biochemical control rather than direct mechanical control of the genes when situated at lower band numbers. We have previously suggested that small RNAs could be important in MSC self-renewal and this is supportive of a biochemical rather than mechanical control mechanism [5].

However, on our osteogenic, NSQ50 surface, highly significant changes in chromosomal positioning and more significant telomeric changes in regulation were noted. We observed expression of the osteogenic gene Runx2 (mapped on 6p21 – i.e. large chromosome, telomeric) but reduced expression of the soft tissue markers. Interestingly, the changes in telomeric gene down-regulations, in MSCs on the NSQ50 topography and the movement of larger territories may contribute to osteogenic differentiation [33].

Literature review provides evidence further supporting that direct mechanotransduction could be a significant part of osteogenesis as a number of significant osteospecific genes (osteocalcin at 1q25–31, osteopontin at 4q22, osteonectin at 5q31 and alkaline phosphatase at 2q37) are located at the more telomeric band positions of the larger chromosomes [31,34]. Combined with our new data, this suggests that the Runx2 osteogenic ‘master’ transcription factor and the genes Runx2 regulates are located in tensionally sensitive areas of the genome. This further demonstrates the tension sensitivity of the osteogenic lineage.

## 5 Conclusions

In the current study we have demonstrated the potential of a non-invasive materials approach to understanding mechanisms underlying stem cell growth and differentiation. Analysis of the architectural and molecular effects of the two different nanotopographies on MSC nucleus organisation show modulation of chromosomal positioning and spatially-related gene changes during osteogenesis but not self-renewal. These studies offer new insight into the differentiation of MSCs on different nanotopographies and the implications therein for modulation of skeletal function and activity. The wider implications for stem cell fate regulation in other systems (foetal, embryonic, soft tissues) are under investigation in our laboratories.

## Figures and Tables

**Table 1:** qPCR primer details for SYBR green.

Gene	Forward primer	Reverse primer
ALCAM	ACGATGAGGCAGACGAGATAAGT	CAGCAAGGAGGAGACCAACAAC
GapDH	GTCAGTGGTGGACCTGACCT	ACCTGGTGCTCAGTGTAGCC

**Table 2:** qPCR details for ABI TaqMan assays.

Gene	ABI TaqMan assay ID
RUNX2	Hs00231692_m1
Sox9	Hs00165814_m1
HOP26	Hs_00156390_m1
PParγ	Hs_01115513_m1
GapDH	4352934E

## References

[1] Dalby M.J., Gadegaard N., Tare R., Andar A., Riehle M.O., Herzyk P. (2007). The control of human mesenchymal cell differentiation using nanoscale symmetry and disorder. Nat Mater.

[2] McNamara L.E., Sjostrom T., Burgess K.E., Kim J.J., Liu E., Gordonov S. (2011). Skeletal stem cell physiology on functionally distinct titania nanotopographies. Biomaterials.

[3] Oh S., Brammer K.S., Li Y.S.J., Teng D., Engler A.J., Chien S. (2009). Stem cell fate dictated solely by altered nanotube dimension. Proc Natl Acad Sci U S A.

[4] Park J.Y., Kricka L.J. (2007). Prospects for nano- and microtechnologies in clinical point-of-care testing. Lab Chip.

[5] McMurray R.J., Gadegaard N., Tsimbouri P.M., Burgess K.V., McNamara L.E., Tare R. (2011). Nanoscale surfaces for the long-term maintenance of mesenchymal stem cell phenotype and multipotency. Nat Mater.

[6] Ji L., LaPointe V.L., Evans N.D., Stevens M.M. (2012). Changes in embryonic stem cell colony morphology and early differentiation markers driven by colloidal crystal topographical cues. Eur Cell Mater.

[7] Chen W., Villa-Diaz L.G., Sun Y., Weng S., Kim J.K., Lam R.H. (2012). Nanotopography influences adhesion, spreading, and self-renewal of human embryonic stem cells. ACS Nano.

[8] Muraglia A., Cancedda R., Quarto R. (2000). Clonal mesenchymal progenitors from human bone marrow differentiate in vitro according to a hierarchical model. J Cell Sci.

[9] Engler A.J., Sen S., Sweeney H.L., Discher D.E. (2006). Matrix elasticity directs stem cell lineage specification. Cell.

[10] Narita Y., Yamawaki A., Kagami H., Ueda M., Ueda Y. (2008). Effects of transforming growth factor-beta 1 and ascorbic acid on differentiation of human bone-marrow-derived mesenchymal stem cells into smooth muscle cell lineage. Cell Tissue Res.

[11] Pittenger M.F., Mackay A.M., Beck S.C., Jaiswal R.K., Douglas R., Mosca J.D. (1999). Multilineage potential of adult human mesenchymal stem cells. Science.

[12] McBeath R., Pirone D.M., Nelson C.M., Bhadriraju K., Chen C.S. (2004). Cell shape, cytoskeletal tension, and RhoA regulate stem cell lineage commitment. Dev Cell.

[13] Kilian K.A., Bugarija B., Lahn B.T., Mrksich M. (2010). Geometric cues for directing the differentiation of mesenchymal stem cells. Proc Natl Acad Sci U S A.

[14] Thomas T.E., Abraham S.J., Lansdorp P.M. (2002). Flow cytometry and immunoselection of human stem cells. Methods Mol Med.

[15] Gilbert P.M., Havenstrite K.L., Magnusson K.E., Sacco A., Leonardi N.A., Kraft P. (2010). Substrate elasticity regulates skeletal muscle stem cell self-renewal in culture. Science.

[16] Tsimbouri P.M., McMurray R.J., Burgess K.V., Alakpa E.V., Reynolds P.M., Murawski K. (2012). Using nanotopography and metabolomics to identify biochemical effectors of multipotency. ACS Nano.

[17] Wang N., Suo Z. (2005). Long-distance propagation of forces in a cell. Biochem Biophys Res Commun.

[18] Wang N., Butler J.P., Ingber D.E. (1993). Mechanotransduction across the cell surface and through the cytoskeleton. Science.

[19] Ingber D.E. (1997). Integrins, tensegrity, and mechanotransduction. Gravit Space Biol Bull.

[20] Lin F., Worman H.J. (1993). Structural organization of the human gene encoding nuclear lamin A and nuclear lamin C. J Biol Chem.

[21] Lin F., Worman H. (1995). Structural organization of the human gene (LMNB1) encoding nuclear lamin B1. Genomics.

[22] Biamonti G., Giacca M., Perini G., Contreas G., Zentilin L., Weighardt F. (1992). The gene for a novel human lamin maps at a highly transcribed locus of chromosome 19 which replicates at the onset of S-phase. Mol Cell Biol.

[23] Moir R.D., Montag-Lowy M., Goldman R.D. (1994). Dynamic properties of nuclear lamins: lamin B is associated with sites of DNA replication. J Cell Biol.

[24] Moir R.D., Spann T.P., Herrmann H., Goldman R.D. (2000). Disruption of nuclear lamin organization blocks the elongation phase of DNA replication. J Cell Biol.

[25] Gadegaard N., Mosler M., Larsen M.B. (2003). Biomimetic polymer nanostructures by injection molding. Macromol Mater Eng.

[26] Livak K.J., Schmittgen T.D. (2001). Analysis of relative gene expression data using real-time quantitative PCR and the 2(−Delta Delta *C*(*T*)) method. Methods.

[27] Huang J., Grater S.V., Corbellini F., Rinck S., Bock E., Kemkemer R. (2009). Impact of order and disorder in RGD nanopatterns on cell adhesion. Nano Lett.

[28] Franceschi R.T., Xiao G., Jiang D., Gopalakrishnan R., Yang S., Reith E. (2003). Multiple signaling pathways converge on the Cbfa1/Runx2 transcription factor to regulate osteoblast differentiation. Connect Tissue Res.

[29] Ingber D.E. (1993). Cellular tensegrity: defining new rules of biological design that govern the cytoskeleton. J Cell Sci.

[30] Ingber D.E. (2003). Tensegrity II. How structural networks influence cellular information processing networks. J Cell Sci.

[31] McNamara L.E., Burchmore R., Riehle M.O., Herzyk P., Biggs M.J., Wilkinson C.D. (2012). The role of microtopography in cellular mechanotransduction. Biomaterials.

[32] Mehta I., Amira M., Harvey A., Bridger J. (2010). Rapid chromosome territory relocation by nuclear motor activity in response to serum removal in primary human fibroblasts. Genome Biol.

[33] Reddy K.L., Zullo J.M., Bertolino E., Singh H. (2008). Transcriptional repression mediated by repositioning of genes to the nuclear lamina. Nature.

[34] Chen D., Zhao M., Mundy G.R. (2004). Bone morphogenetic proteins. Growth Factor.

