# Supplementary material for: A genomics approach in determining nanotopographical effects on MSC phenotype
Source: Biomaterials. 2013 Mar;34(9):2177–84. doi: 10.1016/j.biomaterials.2012.12.019 (PMC3573234; doi:10.1016/j.biomaterials.2012.12.019)
Supplement: Supplementary file 1 [file mmc1.docx]

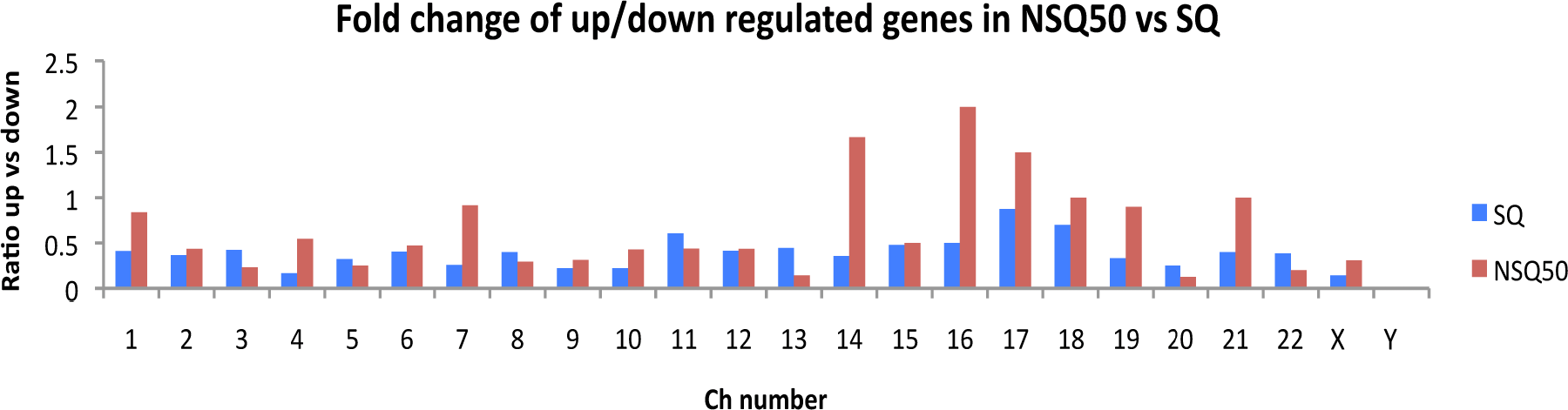


*Supplementary Figure 1.* No of gene up-or down-regulations deriving from Microarray analysis. Microarray was used to select chromosomes that showed either few or many transcript abundance changes. Overall NSQ50 appeared to have the most changes indicating the higher impact this topography has on the MSC gene expression/ cell growth.

*
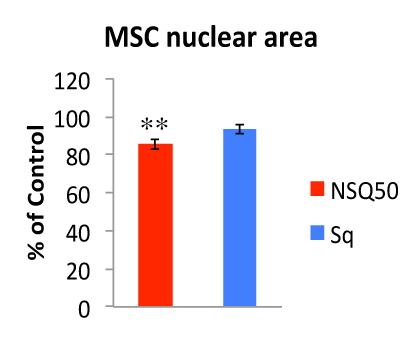
*

*Supplementary Figure 2.* Graph shows mean nuclear area ±std as % of control. MSCs on NSQ50 appeared to have a smaller nuclear area as compared to MSCs on the SQ nanotopography and Flat control. Comparison was done by ANOVA **p*<0.05,***p*<0.01, ****p*<0.001, *n*=120. Note: markings above the error bars denote comparison to flat control.


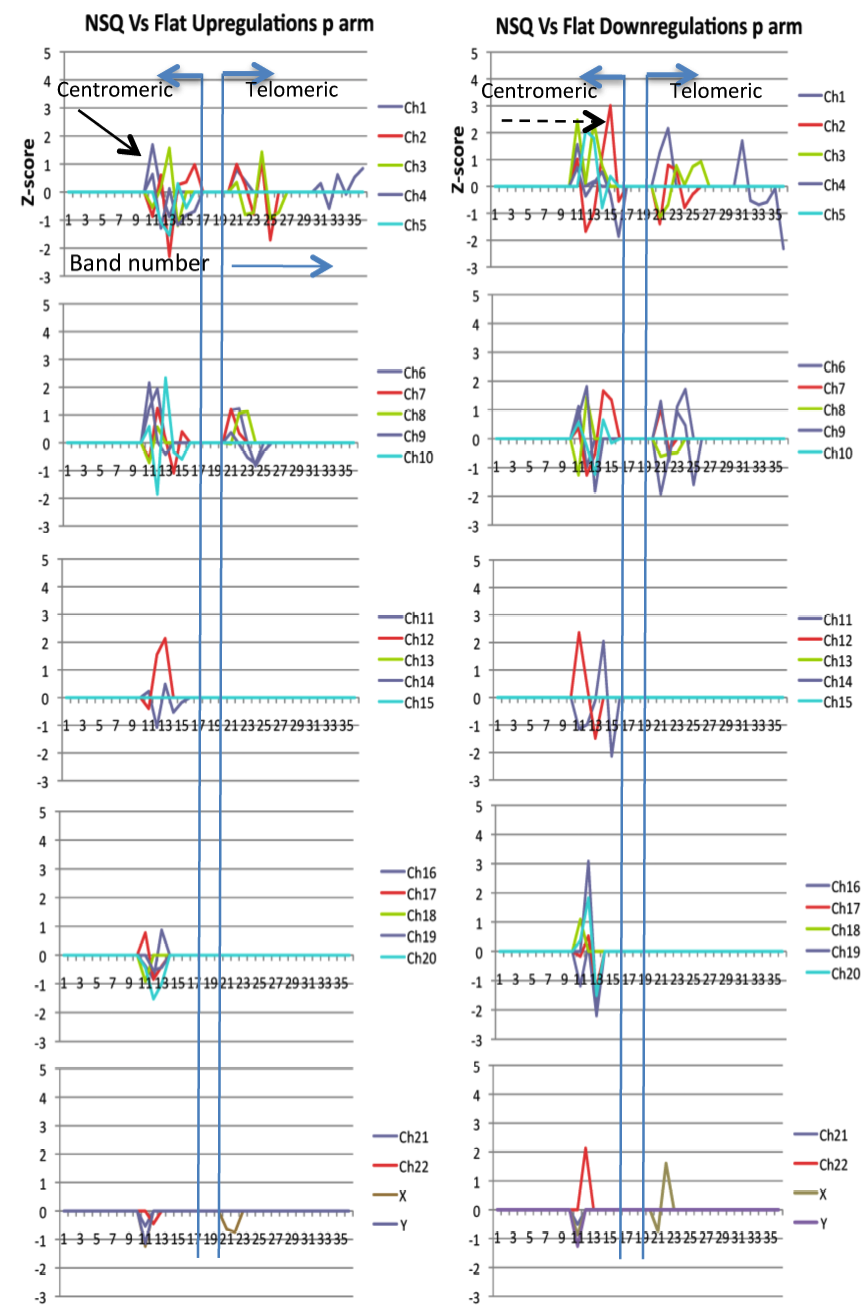


*Supplementary figure 3*. NSQ50 Z-score analysis of gene activity associated with p arm band number. The graphs show significant differences from expected changes based on analysis of 1600 iterations of a ‘window’ of genes that were selected using a threshold of 15% for the false discovery rate. Data is shown NSQ50:Flat (up-regulations/down-regulations refer to the NSQ50 topography, relative to the Flat surface). For the largest chromosomes (1-5), analysis showed points of loss of up-regulation (⭢) and many more substantial gains of down-regulation (⭬ ) at predominantly the centromeric regions (bands 11-15) and less at the telomeric regions (band 21 and above). Profiles were similar for chromosomes 6-10. Interestingly, there were a large number of ‘gains’ of gene down-regulation associated with ‘loss’ of gene up-regulation at the centromeric regions of chromosomes 11 and above.


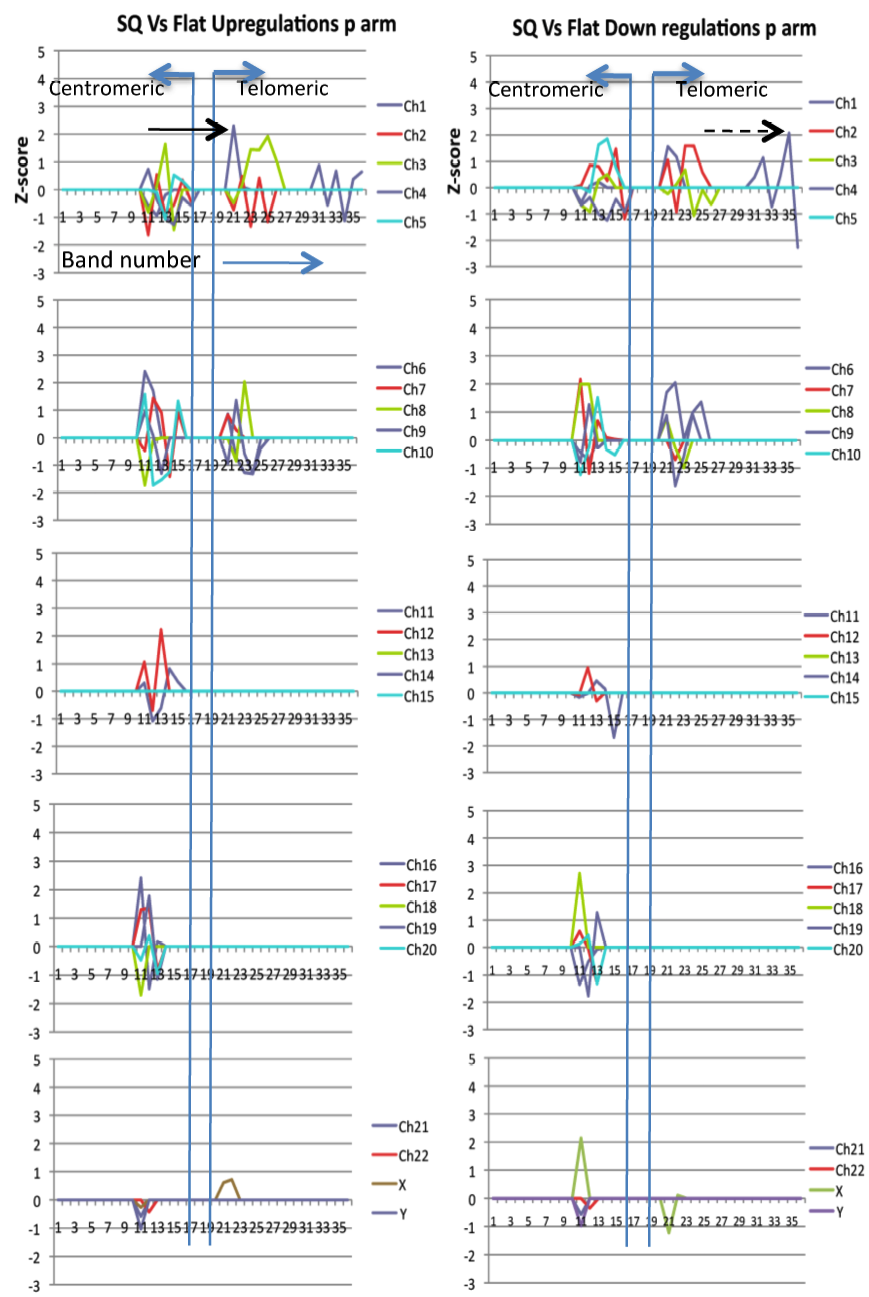


*Supplementary figure 4.* SQ Z-score analysis of gene activity associated with p arm band number. The graphs show significant differences from expected changes based on analysis of 1600 iterations of a ‘window’ of genes that were selected using a threshold of 15% for the false discovery rate. Data is shown SQ:Flat (up-regulations/down-regulations refer to the SQ topography, relative to the Flat surface). For the largest chromosomes (1-5), analysis showed points of gain of up-regulation (⭢) and many more losses of down-regulation (⭬) at the telomeric regions (band 20 and above). Interestingly, there were a large number of ‘gains’ of gene up-regulation associated with ‘loss’ of gene down-regulation at the centromeric regions (band 10-15) of chromosomes 6-15. There were a large number of ‘gains’ of gene down-regulation associated with ‘loss’ of gene up-regulation at the centromeric regions of chromosomes 16 and above.
